# Longitudinal Analyses of Cerebrospinal Fluid α-Synuclein in Prodromal and Early Parkinson’s Disease

**DOI:** 10.1002/mds.27806

**Published:** 2019-07-30

**Authors:** Brit Mollenhauer, Chelsea J. Caspell-Garcia, Christopher S. Coffey, Peggy Taylor, Andy Singleton, Leslie M. Shaw, John Q. Trojanowski, Mark Frasier, Tanya Simuni, Alex Iranzo, Wolfgang Oertel, Andrew Siderowf, Daniel Weintraub, John Seibyl, Arthur W. Toga, Caroline M. Tanner, Karl Kieburtz, Lana M. Chahine, Kenneth Marek, Douglas Galasko

**Affiliations:** 1 Department of Neurology, University Medical Center Goettingen, Göttingen, Germany; and Paracelsus-Elena Klinik, Kassel, Germany; 2 Department of Biostatistics, College of Public Health, University of Iowa, Iowa City, Iowa, USA; 3 BioLegend Inc., San Diego, California, USA; 4 Molecular Genetics Section, Laboratory of Neurogenetics, National Institute on Aging, National Institutes of Health, Bethesda, Maryland, USA; 5 Department of Pathology & Laboratory Medicine, Perelman School of Medicine, University of Pennsylvania, Philadelphia, Pennsylvania, USA; 6 Center for Neurodegenerative Disease Research, Institute on Aging, Perelman School of Medicine, University of Pennsylvania, Philadelphia, Pennsylvania, USA; 7 Morris K. Udall Center of Excellence for Parkinson’s Disease Research, Perelman School of Medicine, University of Pennsylvania, Philadelphia, Pennsylvania, USA; 8 The Michael J. Fox Foundation for Parkinson’s Research, New York, New York, USA; 9 Parkinson’s Disease and Movement Disorders Center, Northwestern University Feinberg School of Medicine, Chicago, Illinois, USA; 10 Neurological Service, Hospital Clinic de Barcelona, Barcelona, Spain; 11 Department of Neurology, Philipps University Marburg, Marburg, Germany; 12 Department of Neurology Perelman School of Medicine, University of Pennsylvania, Philadelphia, Pennsylvania, USA; 13 Institute for Neurodegenerative Disorders, New Haven, Connecticut, USA; 14 University of Southern California, Laboratory of Neuro Imaging, Los Angeles, California, USA; 15 Department of Neurology, University of California San Francisco, San Francisco, California, USA; 16 Clinical Trials Coordination Center, University of Rochester Medical Center, Rochester, New York, USA; 17 Department of Neurology, University of Pittsburgh, Pittsburgh, Pennsylvania, USA; 18 Department of Neurosciences, University of California, San Diego, San Diego, California, USA

**Keywords:** cohort studies, outcome research, Parkinson’s disease/parkinsonism

## Abstract

**Background:**

Aggregation of α-synuclein is central to the pathophysiology of PD. Biomarkers related to α-synuclein may be informative for PD diagnosis/progression.

**Objectives:**

To analyze α-synuclein in CSF in drug-naïve PD, healthy controls, and prodromal PD in the Parkinson’s Progression Markers Initiative.

**Methods:**

Over up to 36-month follow-up, CSF total α-synuclein and its association with MDS-UPDRS motor scores, cognitive assessments, and dopamine transporter imaging were assessed.

**Results:**

The inception cohort included PD (n = 376; age [mean {standard deviation} years]: 61.7 [9.62]), healthy controls (n = 173; age, 60.9 [11.3]), hyposmics (n = 16; age, 68.3 [6.15]), and idiopathic rapid eye movement sleep behavior disorder (n = 32; age, 69.3 [4.83]). Baseline CSF α-synuclein was lower in manifest and prodromal PD versus healthy controls. Longitudinal α-synuclein decreased significantly in PD at 24 and 36 months, did not change in prodromal PD over 12 months, and trended toward an increase in healthy controls. The decrease in PD was not shown when CSF samples with high hemoglobin concentration were removed from the analysis. CSF α-synuclein changes did not correlate with longitudinal MDS-UPDRS motor scores or dopamine transporter scan.

**Conclusions:**

CSF α-synuclein decreases early in the disease, preceding motor PD. CSF α-synuclein does not correlate with progression and therefore does not reflect ongoing dopaminergic neurodegeneration. Decreased CSF α-synuclein may be an indirect index of changes in the balance between α-synuclein secretion, solubility, or aggregation in the brain, reflecting its overall turnover. Additional biomarkers more directly related to α-synuclein pathophysiology and disease progression and other markers to be identified by, for example, proteomics and metabolomics are needed.

Clinical trials for Parkinson’s disease (PD) are currently examining putative neuroprotective agents, but are hampered by the lack of biomarkers that measure key pathophysiological processes. Intracellular aggregation and intercellular spread of pathological forms of α-synuclein (α-syn) are central to the progressive neurodegeneration of PD.^1^

Levels of α-syn in cerebrospinal fluid (CSF) are decreased in PD and other synucleinopathies^2–5^ and may serve as a marker to assist in diagnosis and prognosticating progression. We recently reported that CSF α-syn levels in de novo PD showed minimal change over 12 months.^6^ Longitudinal changes in CSF α-syn and other biomarkers have been examined in other PD cohorts for up to 2 years with discrepant findings.^7,8^ Subject selection, preanalytical factors, and different assays may have contributed to discrepancies (see Discussion). Studies across neurodegenerative disorders indicate that neurodegeneration and biomarker changes start long before the onset of clinical symptoms. Characterizing the longitudinal dynamics of CSF α-syn during prodromal stages and after motor PD begins may advance our understanding of how the spread of α-syn contributes to progression, and can provide benchmark data for the design and interpretation of current and upcoming disease-modifying clinical trials for PD.

We therefore analyzed the levels of total α-syn in longitudinal CSF samples of PD participants and healthy controls (HCs), and in a cohort of prodromal PD. We did not measure subspecies or posttranslationally modified forms of α-syn. We hypothesized that CSF α-syn would change over 36 months with PD progression, that decreased levels would be present in prodromal PD, and that CSF α-syn would correlate with clinical measures or imaging indices of progression.

## Patients and Methods

### Participants

The PPMI (Parkinson’s Progression Markers Initiative) is an ongoing, prospective, longitudinal, observational, international multicenter study that aims to identify biomarkers for the progression of PD. As described,^5,9^ newly diagnosed, drug-naïve PD patients (N = 423) and age- and sex-matched HCs (N = 196) were included (http://ppmi-info.org/study-design). Inclusion and exclusion criteria have been published elsewhere.^9^ Briefly, inclusion criteria for PD participants were the following: (1) aged >30 years; (2) presence of two of the following: bradykinesia, rigidity, and resting tremor or an asymmetric resting tremor or asymmetric bradykinesia; (3) diagnosis made within the last 24 months; (4) PD drug naïvety, and (5) dopamine transporter (DaT) deficit in the putamen on 123-I Ioflupane DaT imaging by central reading.

Between July 2013 and March 2015 participants with isolated (iRBD) rapid eye movement (REM) sleep behavior disorder (RBD) or isolated hyposmia were recruited in PPMI centers for the prodromal part of PPMI. iRBD participants met the following criteria: (1) men or women aged ≥60 years and (2) confirmation of RBD by polysomnography (PSG) with central reading (details below) and/or clinical diagnosis of RBD by the site investigator, including existing PSG. Central PSG interpretation^10^ was based on the following criteria: (1) 18% of any electromyography (EMG) activity in *m. mentalis*, 32% of any EMG activity in mentalis and flexor digitorum superficialis (FDS; in 3-second bins); (2) 27% of any EMG activity in *m. mentalis* and 32% of any EMG activity in *m. mentalis* and FDS (in 30-second bins). In 2 cases, a central PSG reading was not available because of technical difficulties with electronic PSG transfer, but these participants had a clinical diagnosis of iRBD by the site investigator, including previous PSG, and also had to show decreased DaT imaging.

Hyposmic participants were aged ≥60 years with olfaction at or below the 10th percentile by age and sex, as determined by the University of Pennsylvania Smell Identification Test (UPSIT). All iRBD and hyposmic participants also required confirmation from the imaging core at the Institute for Neurodegenerative Disorders that screening DaTscan (or vesicular monoamine transporter type 2/PET scan for sites where DaTscan is not available) was read as eligible. Around 80% of the prodromal participants were selected with a DaT deficit similar to participants with early PD, and 20% were selected with no DaT deficit. Prodromal subjects without DaT deficit were similar in age, sex, and risk profile to those with mild-to-moderate DaT deficit. Exclusion criteria can be found in the study protocol at http://www.ppmi-info.org/study-design/research-documentsand-sops/.

This article is based on α-syn analyses from CSF samples obtained from PD and HCs at baseline and 6-, 12-, 24-, and 36-month visits and for prodromal subjects at baseline and 6- and 12-month visits; overall data were downloaded December 4, 2017 from the PPMI database (www.ppmi-info.org).

### Standard Protocol Approvals, Registrations, and Patient Consent

Approval was received from the ethical standards committee on human experimentation for all human participants. Written informed consent for research was obtained from all study participants. The study is registered with clinicaltrials.gov as NCT01141023.

### CSF Sample Collection and Analysis

CSF was collected using standardized lumbar puncture procedures. Sample handling, shipment, and storage were carried out as described^5^ and according to the PPMI biologics manual (http://ppmi-info.org). Aliquots of 0.5 mL of frozen CSF were used by BioLegend (Cambridge, MA) to measure CSF hemoglobin levels and CSF total α-syn with a sandwich-type immunoassay (BioLegend, San Diego, CA, formerly Covance). In the analyses below, we excluded three CSF values in PD subjects as outliers: all were >5,000 pg/mL (which greatly exceeded the 95% confidence limit for the range of all PD CSF α-syn data), and in all 3 subjects, subsequent longitudinal CSF levels of α-syn were substantially (>50%) lower.

### Clinical Assessment Measures

The clinical assessment battery is described on the PPMI website and has been published previously.^11^ In brief, motor assessment used the revised UPDRS published by the International Parkinson and Movement Disorder Society (MDS-UPDRS III and total score).^12^ Use of medications for PD was recorded at each visit after baseline assessment and is expressed as levodopa equivalent doses (LEDs)^13^ and stratified according to LED subtotal from dopamine replacement or dopamine agonists.

Cognitive testing included the Montreal Cognitive Assessment (MoCA) and psychometric tests of memory (Hopkins Verbal Learning Test-revised; HVLT-R), processing speed/attention (Symbol Digit Modality Test; SDMT), executive function/working memory (Wechsler Memory Scale–Third Edition Letter-Number Sequencing [LNS] test), and visuospatial abilities (Benton Judgment of Line Orientation [BJLO] test).^14^ The REM Sleep Behavior Disorder Screening Questionnaire (RBDSQ) was used to assess subjectively reported symptoms of RBD.^15^

### Dopamine Single-Photon Emission Computing Tomography Imaging

Dopamine imaging was performed by DaTscan using standardized methods.^9^ Quantitative DaTscan measures in striatal binding ratio (SBR) of caudate, putamen, or striatal uptake were used in our analyses.

### Genetic Variables

To examine whether selected genetic variants were associated with CSF biomarkers, we used data for *APOE* genotypes, *MAPT*, and single-nucleotide polymorphisms related to *SNCA* (i.e., rs3910105 and rs356181), measured by the PPMI Genetics Core.^16^

*SNCA* transcripts were analyzed as documented in the Laboratory of Neuroimaging (LONI) database (https://ida.loni.usc.edu/pages/access/studyData.jsp?categoryId=7&subCategoryId=52) by assaying transcript counts in human blood in a high-precision nanoString gene expression assay. PAXgene tubes (Qiagen, Valencia, CA) were collected by venipuncture according to standardized protocols (http://www.ppmi-info.org/study-design/research-documents-andsops/), incubated at room temperature for 24 hours, frozen, and shipped on dry ice. RNA extraction, followed the PAXgene procedure and quality control, was performed using the RNA Integrity Number package.^17^ The *SNCA* probes used target the boundaries of exon 3 and exon 4 (termed E3E4-SNCA), transcripts specifically with a long 3-untranslated region (3UTR) region (termed 3UTR-1 and 3UTR2-SNCA), transcripts that skip exon 5 (termed E4E6-SNCA), or the rate shot SNCA-007 transcript isoform (Enseml ID ENST00000506691) that comprises exons 1–4.

### Statistical Analysis

Statistical analyses were performed using SAS software (version 9.4; SAS Institute Inc., Cary, NC) on data retrieved from the PPMI data portal at the LONI at the University of Southern California. All tests performed using CSF α-syn were rank-based. *t* tests or chi-square were used to compare baseline demographic and clinical variables in participants with longitudinal CSF data versus participants who only had baseline CSF data; these comparisons were performed separately in all four groups. Repeated-measures linear mixed models were used to test for changes over time in CSF α-syn levels separately by group. In addition, repeated-measures linear mixed models were used to examine longitudinal relationships between CSF α-syn levels and PD medication use.

Simple linear models were used to analyze potential baseline predictors of baseline CSF α-syn, separately in PD and HCs. First, the univariate relationship between each predictor and CSF α-syn level was examined. Then, any variables that had univariate associations with a *P* values <0.2 were included in a multivariable model. Finally, a backward selection process was used to remove variables individually until all variables remaining in the model were significant at the 0.1 level.

Spearman rank-correlation coefficients between changes in CSF α-syn levels and changes in clinical progression variables and changes in DaTscan measures were reported, and also for SNCA transcript information and CSF α-syn levels. Kruskal-Wallis H tests were used to test for associations between CSF α-syn levels and genetic variables.

## Results

### Demographic and Clinical Data

The study enrolled 423 PD, 196 HC, 39 iRBD, and 26 hyposmic participants. From these participants, 376 PD, 173 HC, 32 prodromal iRBD, and 16 prodromal hyposmic participants had complete data, including CSF; their demographic and clinical data at baseline visits are shown in Table 1A and Table 1B. Comparison of participants with CSF baseline and longitudinal data versus those with baseline data showed that PD participants who dropped out after baseline had slightly worse cognitive performance shown on HVLT (*P* = 0.039), SDMT (*P* < 0.001), LNS (*P* = 0.031), and in BJLO (*P* = 0.002). Prodromal hyposmic participants with baseline data had worse cognitive performance (on HVLT, *P* = 0.0007; SDMT, *P* = 0.045) and lower mean caudate SBR values (*P* = 0.011) on DaTscan. iRBD participants with milder iRBD by RBDSQ (<6) were more likely to drop out after baseline assessment (*P* = 0.029; data not shown).

### Baseline and Longitudinal CSF α-syn Values

**FIG. 1.**
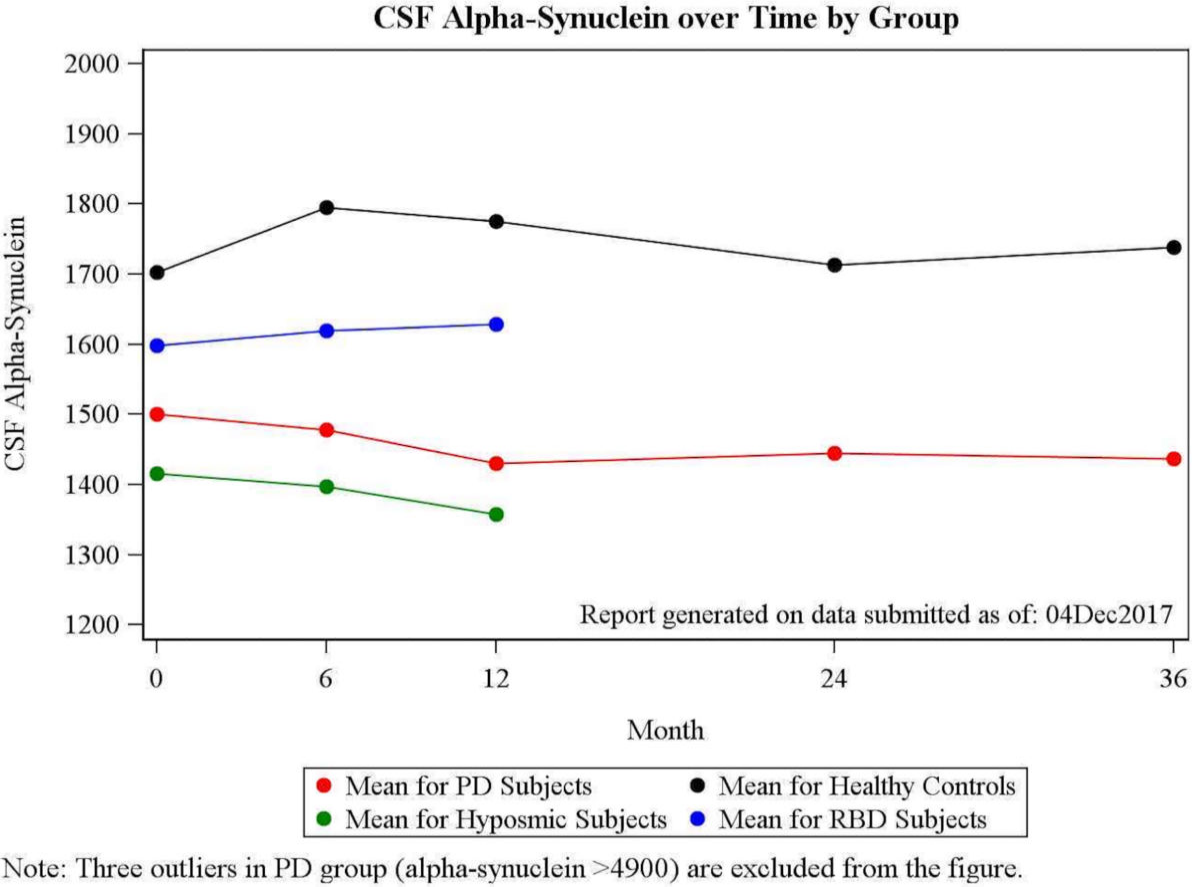
Mean CSF α-syn levels at each visit in control, PD, and prodromal groups.

CSF α-syn levels were significantly lower in PD compared to HC across all visits (*P* < 0.0001). Changes in CSF α-syn in PD, controls, and both prodromal cohorts over time are shown in Table 2 and Figure 1. CSF total α-syn levels in PD decreased slightly from baseline to 36 months (*P* = 0.032), whereas levels did not change in the control group (*P* = 0.054; Table 2). Longitudinal changes were not significant in analyses restricted to the 185 PD and 86 HC samples with low hemoglobin concentrations (<200 ng/mL; *P* = 0.196).

Among prodromal groups, the hyposmic participants showed the lowest mean CSF α-syn levels, whereas iRBD participants had intermediate levels between HCs and PD. In both prodromal groups, CSF α-syn remained relatively stable over the study interval from baseline to 6 and 12 months (*P* = 0.915 for hyposmic and *P* = 0.714 for prodromal RBD participants).

### Baseline Predictors of Change in CSF α-syn and Correlation With Clinical Progression Variables

In multivariate regression analysis, older age (*P* = 0.007), and height (*P* = 0.002, but not body mass index [BMI]) were significant predictors of baseline CSF α-syn in PD participants (data not shown). Changes in CSF α-syn were not related to changes in MDS-UPDRS III, MoCA, and DaTscan values (*P* > 0.05) in PD, and had a relationship to MoCA changes in HC over 36 months (*P* = 0.021; Table 3). In the iRBD group, there was a significant negative correlation between CSF α-syn and MDS-UPDRS III over 12 months (*P* = 0.037).

To examine different phenotypes, we analyzed the correlations of change of CSF α-syn in PD participants showing hyposmia (by UPSIT <25) and REM sleep behavior symptoms (RBDSQ >6) and found no significant correlation between clinical progression and CSF α-syn in these subgroups (data not shown). In PPMI PD subjects, we previously observed a greater decrease in CSF α-syn levels over 12 months in subjects who took dopamine medications, with a weak relationship with LED.^6^ We again found a longitudinal relationship between CSF α-syn and LED based on dopamine replacement (*P* = 0.016), but it lost significance when we excluded samples with hemoglobin <200 ng/mL (*P* = 0.361 and 0.083; Table 4).

### Association of CSF α-syn With Genetics and SNCA Transcripts

Genetic variants in *APOE* e4, *MAPT*, and polymorphisms in the *SNCA* gene (*SNCA* rs356181 and rs3910105) were not associated with baseline or longitudinal change of CSF α-syn in PD and HCs (*P* > 0.05). Another recent study found that a polygenic hazard score also showed no association with CSF α-syn levels.^18^ *SNCA* transcripts were not associated with baseline and longitudinal α-syn in PD or in HC (data not shown).

### Variability of α-syn Measurements Between 2013 and 2016

A subset of PD participants and HCs had CSF α-syn measured in 2013 and again (from different CSF aliquots, but using the same enzyme-linked immunosorbent assay [ELISA]) in 2016. Levels from both analyses were strongly correlated (Spearmen rho = 0.71; *P* < 0.001), with a systematic shift toward lower values of α-syn in the 2016 analyses relative to those of 2013 (data not shown). The shift may be attributed to preanalytical factors in CSF sample handling (e.g., aliquoting or gradient effects or long-term freezing effect) or analytical/assay factors, which can occur with the manual performance of multiple 96-well ELISA plates. Ongoing studies of α-syn measurement, including mass spectrometry, will address some of these issues.^19^

## Discussion

Longitudinal changes in CSF α-syn and other biomarkers in PD have been examined in other cohorts for up to 2 years: two studies show increasing CSF levels over time,^7,8^ one reported a decrease^20^ and another more recent study showed no longitudinal effects in a small cohort.^21^ Subject characteristics, preanalytical factors, and different assays may have contributed to these discrepancies. Discrepant dynamics of CSF α-syn were found in samples from the DATAOP (Deprenyl and Tocopherol Antioxidant Therapy of Parkinsonism) trial, in analyses that used different assays and inclusion criteria.^20^ One study that reported an increase over time only included samples from participants with diagnostic likelihood of clinical PD estimated at 90% to 100%, whereas another study also excluded subjects with other neurological disorders identified during follow-up.^22^ In contrast, the study reporting a decrease included the entire DATATOP cohort without selection.

The strengths of the PPMI include multicenter recruitment, clinical, biosample, and imaging standardization, high rates of follow-up, and inclusion of prodromal patients at risk for PD. We have now extended the interval of follow-up for CSF biomarkers in PD and HCs in the PPMI to 36 months compared to our previous analyses.^6^ To assess how early in the disease course CSF α-syn may decrease, we also evaluated levels in prodromal participants with hyposmia and iRBD, both of which carry high risk for PD or related disorders.^23,24^ Overall, CSF α-syn decreased longitudinally in PD and increased slightly (nonsignificant) in HCs over 36 months. The magnitude of change was small and was no longer significant when around 50% of CSF samples with hemoglobin contamination were removed. Because of the high abundance of α-syn in blood, blood contamination during lumbar puncture impacts CSF α-syn. At baseline, the PD-HC differences in CSF α-syn remained significant even when samples with high hemoglobin concentrations (>200 ng/mL) were included. Therefore, the loss of longitudinal significance with exclusion for high hemoglobin concentrations could be explained by the lower numbers of PD subjects at baseline (n = 187 vs. n = 376), but could also indicate no longitudinal change of CSF α-syn in PD.

The 10% to 15% decrease in CSF α-syn in PD versus controls is a consistent finding across cohorts^25^ with some variability; one earlier study that included iRBD had only a small number of controls.^26^ Despite marked overlap of individual values with HC, mean levels in PPMI PD subjects were significantly lower than HC across all visits. The reason for the decrease remains unclear. One explanation is that CSF α-syn is decreased because of intracellular aggregation of α-syn in the brain. A recent review^27^ summarizes the different pathways involved in the degradation of intracellular α-syn that includes chaperone-mediated autophagy, endosomal, and proteasomal degradation as well as macroautophagy. Extracellular α-syn may represent other clearance pathways and is subject to proteolysis by extracellular proteases, such as neurosin, that has been detected in CSF^28^ and that inversely correlated with α-syn accumulation in brains with dementia with Lewy bodies (DLB).^29^

Our findings that subjects with likely prodromal PD (iRBD and hyposmic subjects with 80% of pathological DaTscan) already have decreased CSF α-syn is consistent with significant pathology being present during these prodromal stages, analogous to the decrease in CSF amyloid beta 42 in Alzheimer’s disease.^30^ iRBD is a highly specific prodromal condition with a high conversion rate (>80%) to an α-syn aggregation disorder after 16 years.^31^ PD subjects with RBD may have a more aggressive form of PD with cognitive decline.^32^ iRBD may progress to more aggressive α-syn aggregation disorders (i.e., to DLB) and—rarely—to MSA. Greater neurodegeneration may contribute to the finding of higher levels in the iRBD than the PD and hyposmia groups.

It is unclear whether the decrease of CSF α-syn develops even earlier during prodromal PD or may represent a trait that is a risk factor for PD. In the PPMI, CSF α-syn levels in HC subjects are followed longitudinally to see whether those with lower levels may develop PD later.

The lack of a relationship between CSF α-syn and genetic risk factors or *SNCA* transcripts supports a state, rather than a trait, marker. How α-syn gets into CSF is incompletely understood, although a recent study indicates that neuronal activity, particularly at excitatory synapses, is a major contributor to its release.^33^ In contrast to PD, levels of α-syn are markedly increased in CSF in Creutzfeldt-Jakob disease, where rapid and progressive neuronal death occurs.^34^

The reasons for CSF α-syn variability remain to be determined. One explanation could be partly attributed to misdiagnoses that are not excluded in this cohort and that are reported to be a problem among de novo PD subjects.^35^ To date, based on thorough neurological judgement at each visit, there are three misdiagnoses among the PD subjects analyzed here: 2 were diagnosed with MSA (1 with autopsy confirmation) and 1 with corticobasal degeneration. Further clinical follow-up, and the approved brain donation program in the PPMI as well as future biomarker approaches, for example, the ratio of α-syn/tau protein,^36^ neurofilament light chain,^37^ or others could help to distinguish PD from atypical PD syndromes.

The decrease of CSF α-syn in PD over 36 months did not correlate with progression of motor and nonmotor symptoms, or with a decrease of dopamine transporter signal, both robust indices of PD progression. Therefore, the events that result in decreased CSF α-syn do not appear to directly drive PD progression. We confirmed the earlier association of symptomatic medication with greater decline in CSF α-syn in PD, for unclear reasons. We could not identify other predictors of changes in CSF besides age and possibly BMI.

Although we observed a significant decrease of CSF α-syn in PD over 36 months’ follow-up, and levels tended to be stably decreased within patients, CSF α-syn, as measured with the assay used, will not serve as a diagnostic marker for PD and is unlikely to be a sole outcome measure for clinical trials or progression. Substantial overlap between PD and HC groups may result from biological or genetic variability, (co)medication, comorbidities, or other factors. Furthermore, subtypes of PD may reflect different pathophysiological factors, with clinical heterogeneity. Other CSF biomarkers are currently being analyzed in the PPMI, including total and phosphorylated tau protein, and β-amyloid 1–42, reflecting different pathological contributions to cognitive and motor progression.^38^ Further progress in diagnostic and progression biomarkers will benefit from analysis of abnormal forms of α-syn^39–42^ and of novel biomarkers identified through methods such as proteomics and metabolomics.

## Figures and Tables

**Table 1A.** Baseline demographics and clinical characteristics

Variable	PD Participants (N = 376)	Healthy Controls (N = 173)	Prodromal Hyposmic (N = 16)	Prodromal iRBD (N = 32)
Age Mean (SD)	61.7 (9.62)	60.9 (11.3)	68.3 (6.15)	69.3 (4.83)
(min, max)	(33, 85)	(31, 84)	(61, 81)	(61, 82)
Missing	0	0	0	0
Sex (%) Men	246 (65.4)	110 (63.6)	10 (62.5)	26 (81.3)
Women	130 (34.6)	63 (36.4)	6 (37.5)	6 (18.8)
Age at PD onset Mean (SD)	59.7 (9.87)	N/A	N/A	N/A
(min, max)	(25, 83)	N/A	N/A	N/A
Missing	0	N/A	N/A	N/A
MDS-UPDRS Part III Mean (SD)	21.0 (8.76)	1.3 (2.29)	2.8 (3.19)	4.5 (3.73)
(min, max)	(4, 51)	(0, 13)	(0, 10)	(0, 15)
Missing	0	1	0	0
UPSIT (%) >25	137 (36.4)	163 (94.2)	2 (12.5)	5 (15.6)
<25	239 (63.6)	10 (5.8)	14 (87.5)	26 (81.3)
Missing	0 (0)	0 (0)	0 (0)	1 (3.1)
RBDSQ (%) <6	235 (62.5)	139 (80.3)	8 (50.0)	2 (6.3)
≥6	138 (36.7)	34 (19.7)	8 (50.0)	30 (93.8)
Missing	3 (0.8)	0 (0)	0 (0)	0 (0)
MoCA Mean (SD)	27.2 (2.29)	28.2 (1.10)	27.7 (1.40)	25.2 (4.36)
(min, max)	(17, 30)	(27, 30)	(25, 30)	(11, 30)
Missing	3	0	0	0
Mean caudate [SBR] Mean (SD)	2.0 (0.56)	3.0 (0.62)	2.5 (0.62)	1.9 (0.44)
(min, max)	(0, 4)	(1, 5)	(2, 4)	(1, 3)
Missing	3	1	0	0
Mean putamen [SBR] Mean (SD)	0.8 (0.28)	2.1 (0.55)	1.4 (0.34)	1.1 (0.33)
(min, max)	(0, 2)	(1, 4)	(1, 2)	(1, 2)
Missing	3	1	0	0
Mean striatum [SBR] Mean (SD)	1.4 (0.40)	2.6 (0.57)	2.0 (0.47)	1.5 (0.36)
(min, max)	(0, 3)	(1, 4)	(1, 3)	(1, 2)
Missing	3	1	0	0

N/A, not applicable.

**TABLE 1B.** Additional baseline clinical characteristics

Variable	PD Participants (N = 376)	Healthy Controls (N = 173)	Prodromal Hyposmic (N = 16)	Prodromal iRBD (N = 32)
MDS-UPDRS total score Mean (SD)	32.3 (13.0)	4.7 (4.37)	10.7 (7.52)	14.9 (7.48)
(min, max)	(7, 70)	(0, 20)	(0, 26)	(4, 31)
Missing	32	5	11	15
Motor subgroup (%) TD	270 (71.8)	N/A	N/A	N/A
PIGD	105 (27.9)	N/A	N/A	N/A
Missing	1 (0.3)	N/A	N/A	N/A
HVLT total recall Mean (SD)	24.6 (4.91)	26.3 (4.51)	24.8 (5.02)	21.3 (5.29)
(min, max)	(9, 36)	(15, 35)	(16, 33)	(9, 33)
Missing	25	26	25	21
HVLT delayed recall Mean (SD)	8.4 (2.51)	9.3 (2.34)	9.2 (2.34)	6.6 (3.38)
(min, max)	(0, 12)	(2, 12)	(5, 12)	(0, 12)
Missing	8	9	9	7
HVLT discrimination recognition Mean (SD)	9.7 (2.66)	10.0 (2.93)	10.8 (0.77)	8.7 (2.48)
(min, max)	(–4, 12)	(–4, 12)	(9, 12)	(2, 12)
Missing	10	10	11	9
SDMT Mean (SD)	41.8 (9.37)	46.8 (10.8)	45.6 (8.14)	30.8 (8.19)
(min, max)	(7, 82)	(20, 83)	(24, 55)	(15, 47)
Missing	42	47	46	31
LNS Mean (SD)	10.7 (2.58)	11.0 (2.55)	10.5 (2.00)	8.9 (3.57)
(min, max)	(2, 20)	(4, 20)	(6, 14)	(3, 17)
Missing	11	11	11	9
BJLO Mean (SD)	12.9 (2.08)	13.1 (2.00)	13.2 (1.87)	11.2 (2.37)
(min, max)	(5, 15)	(4, 15)	(8, 15)	(3, 15)
Missing	13	13	13	11
APOE e4 (%) 0 e4 alleles	254 (67.6)	120 (69.4)	N/A	N/A
1 e4 allele	83 (22.1)	35 (20.2)	N/A	N/A
2 e4 alleles	8 (2.1)	4 (2.3)	N/A	N/A
Missing	31 (8.2)	14 (8.1)	N/A	N/A
SNCA rs3910105 (%) C/C	59 (15.7)	39 (22.5)	N/A	N/A
C/T	175 (46.5)	75 (43.4)	N/A	N/A
T/T	111 (29.5)	49 (28.3)	N/A	N/A
Missing	31 (8.2)	10 (5.8)	N/A	N/A
SNCA rs356181 (%) C/C	104 (27.7)	30 (17.3)	N/A	N/A
C/T	163 (43.4)	85 (49.1)	N/A	N/A
T/T	78 (20.7)	48 (27.7)	N/A	N/A
Missing	31 (8.2)	10 (5.8)	N/A	N/A
MAPT (%) H1/H1	216 (62.6)	103 (63.2)	N/A	N/A
H1/H2	113 (32.8)	53 (32.5)	N/A	N/A
H2/H2	16 (4.6)	7 (4.3)	N/A	N/A
Missing	0 (0)	0 (0)	N/A	N/A

TD, tremor dominant; PIGD, postural instability and gait difficulty; N/A, not applicable.

**Table 2.** CSF α-syn over time by group

Group	Baseline	6 Months	12 Months	24 Months	36 Months	*P* Value (Change Over Time)
PD						**0.032**
N	374	338	325	306	229	
Median	1,374.3	1,313.3	1,313.0	1,324.0	1,344.0	
(min, max)	(432.4, 3,760.0)	(482.3, 4,279.1)	(420.0, 3,685.3)	(336.1, 3,871.4)	(458.8, 3,621.1)	
PD (low hemoglobin^a^)						0.232
N	185	170	160	155	105	
Median	1,320.2	1,306.6	1,328.5	12,924.7	1,297.3	
(min, max)	(487.8, 3,638.3)	(482.3, 4,071.7)	(425.8, 3,685.3)	(356.1, 3,871.4)	(542.8, 3,621.1)	
Controls						0.054
N	173	159	153	135	113	
Median	1,582.4	1,736.0	1,646.6	1,661.0	1,695.9	
(min, max)	(488.6, 4,683.1)	(521.6, 5,153.5)	(517.1, 4,388.6)	(484.6, 4,202.0)	(496.2, 5,034.5)	
Controls (low hemoglobin^a^)						0.531
N	86	77	73	63	51	
Median	1,626.8	1,748.4	1,709.1	1,667.9	1,733.8	
(min, max)	(600.7, 4,139.4)	(521.6, 4,087.9)	(642.1, 4,184.1)	(518.8, 4,202.0)	(496.2, 3,992.8)	
Prodromal hyposmic						0.915
N	16	15	11	—	—	
Median	1,568.4	1,380.3	1,559.8	—	—	
(min, max)	(437.4, 1,937.1)	(447.9, 2,459.3)	(480.4, 1,845.5)	—	—	
Prodromal hyposmic (low hemoglobin^a^)						0.480
N	10	10	6	—	—	
Median	1,284.4	1,016.0	866.3	—	—	
(min, max)	(437.4, 1,937.1)	(447.9, 2,459.3)	(480.4, 1,845.5)	—	—	
Prodromal RBD						0.714
N	32	30	25	—	—	
Median	1,359.4	1,513.5	1,288.6	—	—	
(min, max)	(446.1, 4,325.5)	(613.3, 4,314.7)	(509.7, 4,076.3)	—	—	
Prodromal RBD (low hemoglobin^a^)						0.673
N	21	19	16	—	—	
Median	1,323.2	1,454.8	1,225.9	—	—	
(min, max)	(446.1, 4,325.5)	(613.3, 4,314.7)	(509.7, 4,076.3)	—	—	

*P* values are based on the ranks of the variables.

Three outliers in the PD group (CSF α-syn: >4,900 pg/mL) were excluded from the table.

a Subset of participants with hemoglobin <200 ng/mL at all time points. Excludes participants missing hemoglobin values at one or more time points.

**Table 3.** Correlations of change in CSF α-syn with change in clinical progression variables over time

Variable	Change From Baseline to			
	6 Months	12 Months	24 Months	36 Months
PD participants	(N = 338)	(N = 325)	(N = 306)	(N = 229)
MDS-UPDRS III score	0.022 (*P* = 0.696)	−0.020 (*P* = 0.739)	−0.020 (*P* = 0.758)	−0.020 (*P* = 0.801)
MoCA	—	−0.015 (*P* = 0.791)	−0.005 (*P* = 0.928)	−0.024 (*P* = 0.721)
Mean caudate [SBR]	—	0.020 (*P* = 0.723)	0.098 (*P* = 0.098)	−0.433 (*P* = 0.244)
Mean putamen [SBR]	—	0.006 (*P* = 0.914)	0.026 (*P* = 0.655)	−0.067 (*P* = 0.865)
Mean striatum [SBR]	—	0.013 (*P* = 0.822)	0.082 (*P* = 0.165)	−0.250 (*P* = 0.516)
Healthy controls	—	(N = 153)	(N = 135)	(N = 113)
MDS-UPDRS III score	—	−0.079 (*P* = 0.333)	−0.039 (*P* = 0.656)	−0.184 (*P* = 0.051)
MoCA	—	−0.110 (*P* = 0.176)	−0.043 (*P* = 0.623)	−0.218 (***P* = 0.021**)
Prodromal hyposmic	(N = 15)	(N = 11)	—	—
MDS-UPDRS III score	−0.060 (*P* = 0.833)	−0.494 (*P* = 0.122)	—	—
MoCA	—	0.051 (*P* = 0.882)	—	—
Mean caudate [SBR]	—	0.006 (*P* = 0.987)	—	—
Mean putamen [SBR]	—	−0.127 (*P* = 0.726)	—	—
Mean striatum [SBR]	—	−0.030 (*P* = 0.934)	—	—
Prodromal iRBD	(N = 30)	(N = 25)	—	—
MDS-UPDRS III score	0.029 (*P* = 0.879)	−0.428 (***P* = 0.037**)	—	—
MoCA	—	−0.027 (*P* = 0.899)	—	—
Mean caudate [SBR]	—	−0.058 (*P* = 0.783)	—	—
Mean putamen [SBR]	—	−0.155 (*P* = 0.459)	—	—
Mean striatum [SBR]	—	−0.088 (*P* = 0.674)	—	—

Healthy controls only completed DaTscan at baseline. Three outliers among the PD group (aberrant value of CSF α-syn >4,900 at one time point) were excluded.

**Table 4.** Longitudinal relationship between CSF α-syn and PD medications in PD participants

Variable	PD Participants	
	Estimate (95% CI)	*P* Value
Relationship with CSF α-syn levels PD medication use	4.55 (–38.24, 47.34)	0.834
Total LED	0.055 (−0.014, 0.125)	0.118
LED subtotal: dopamine replacement	0.086 (0.016, 0.156)	**0.016**
LED subtotal: dopamine agonists	−0.155 (−0.341, 0.031)	0.102
Relationship with CSF α-syn levels^a^ PD medication use	9.60 (−50.80, 69.99)	0.754
Total LED	0.007 (−0.080, 0.094)	0.878
LED subtotal: dopamine replacement	0.040 (−0.046, 0.127)	0.361
LED Subtotal: dopamine agonists	−0.292 (−0.560, −0.024)	0.083

*P* values are based on the ranks of CSF α-syn levels.

a Subset of participants with hemoglobin <200 ng/mL at all time points.

Excludes participants missing hemoglobin values at one or more time points and the 3 PD subjects with outlying/aberrant CSF data.

CI, confidence interval.

## Contributors’ Appendix

### Steering Committee

Kenneth Marek, MD (Principal Investigator), Danna Jennings, MD (Olfactory Core, PI); Shirley Lasch, MBA; Caroline Tanner, MD, PhD (Site Investigator, Sleep WG), Tatyana Simuni, MD (Site Investigator), Christopher Coffey, PhD (Statistics Core, PI), Karl Kieburtz, MD, MPH (Clinical Core, PI), Renee Wilson, Werner Poewe, MD (Site Investigator), Brit Mollenhauer, MD (Site Investigator), Tatiana Foroud, PhD (Genetics Coordination Core, PI); Todd Sherer, PhD, Sohini Chowdhury, Mark Frasier, PhD; Catherine Kopil, PhD; Vanessa Arnedo.

### Study Cores: update

*Clinical Coordination Core*: Alice Rudolph, PhD, Cynthia Casaceli, MBA; *Imaging Core*: John Seibyl, MD (Principal Investigator), Susan Mendick, MPH, Norbert Schuff, PhD; *Statistics Core*: Chelsea Caspell, Liz Uribe, Eric Foster, Katherine Gloer, PhD, Jon Yankey, MS; *Bioinformatics Core*: Arthur Toga, PhD (Principal Investigator), Karen Crawford; *BioRepository*: Paola Casalin, Giulia Malferrari; *Bioanalytics Core*: Brit Mollenhauer, MD, Douglas Galasko, MD, MS; *Genetics Core*: Andrew Singleton, PhD (Principal Investigator); *Neuropsychological and Cognitive Assessments*: Keith A. Hawkins, PsyD.

### Site Investigators

David Russell, MD, PhD; Stewart Factor, DO; Penelope Hogarth, MD; David Standaert, MD, PhD; Robert Hauser, MD, MBA; Joseph Jankovic, MD; Matthew Stern, MD; Lana Chahine, MD; James Leverenz, MD; Samuel Frank, MD; Irene Richard, MD; Klaus Seppi, MD; Holly Shill, MD; Hubert Fernandez, MD; Daniela Berg, MD; Isabel Wurster, MD, Douglas Galasko, MD, MS; Zoltan Mari, MD; David Brooks, MD; Nicola Pavese, MD; Paolo Barone, MD, PhD; Stuart Isaacson, MD; Alberto Espay, MD, MSc; Dominic Rowe, MD, PhD; Melanie Brandabur, MD; James Tetrud, MD; Grace Liang, MD; Alex Iranzo, MD (Sleep WG); Eduardo Tolosa, MD (Sleep WG).

### Coordinators

Laura Leary; Cheryl Riordan; Linda Rees, MPH; Alicia Portillo; Art Lenahan; Karen Williams; Stephanie Guthrie, MSN; Ashlee Rawlins; Sherry Harlan; Christine Hunter, RN; Baochan Tran; Abigail Darin; Carly Linder, Marne Baca; Heli Venkov; Cathi-Ann Thomas, RN, MS; Raymond James, RN; Cheryl Deeley, MSN; Courtney Bishop, BS; Fabienne Sprenger, MD; Diana Willeke; Sanja Obradov; Jennifer Mule; Nancy Monahan; Katharina Gauss; Deborah Fontaine, BSN, MS; Shawnees Peacock; Arita McCoy; Becky Dunlop; Bina Shah, BSc; Susan Ainscough; Angela James; Rebecca Silverstein; Kristy Espay; Madelaine Ranola.

### The Parkinson Progression Marker Initiative (PPMI) Executive Steering Committee

Kenneth Marek, MD, Principal Investigator, Danna Jennings, MD, Shirley Lasch; Andrew Siderowf, MD; Caroline Tanner, MD, PhD (Site Investigator); Tatyana Simuni, MD (Site Investigator); Chris Coffey, PhD, (Statistics Core, PI). Karl Kieburtz, MD, MPH (Clinical Core, PI), Emily Flagg, (Clinical Core, Project Manager); Sohini Chowdhury. Steering Committee/Cores: Werner Poewe, MD (Site Investigator); Brit Mollenhauer, MD (Site Investigator); Todd Sherer, PhD, Mark Frasier, PhD, Claire Meunier. Study Cores: Clinical Coordination Core: Alice Rudolph, PhD, Cindy Casaceli. Imaging Core: John Seibyl, MD, Principal Investigator, Susan Mendick, MPH; Norbert Schuff, PhD. Statistics Core: Ying Zhang. Bioinformatics Core: Arthur Toga, PhD, Principal Investigator, Karen Crawford, Laboratory of Neuroimaging (LONI). BioRepository: Alison Ansbach, MS, Principal Investigator; Pas-quale De Blasio, Michele Piovella, BioRep Bioanalytics Core: John Trojanowski, MD, PhD, Principal Investigator, Leslie M. Shaw, PhD, Principal Investigator Genetics Core: Andrew Singleton, PhD, Principal Investigator, Bethesda, MD Neuropsychological and Cognitive Assessments: Keith Hawkins, PsyDMichael J Fox Foundation: Jamie Eberling, PhD, Deborah Brooks Site Investigators and Coordinators: David Russell, MD, PhD, Laura Leary, BS; Stewart Factor, DO, Barbara Sommerfeld, RN, MSN; Penelope Hogarth, MD, Emily Pighetti; Karen Williams; David Standaert, MD, PhD, Stephanie Guthrie; Robert Hauser, MD, Holly Delgado, RN; Joseph Jankovic, MD, Christine Hunter, RN, CCRC; Matthew Stern, MD, Baochan Tran; Jim Leverenz, MD, Marne Baca; Sam Frank, MD, Cathi-Ann Thomas, RN, MS; Irene Richard, MD, Cheryl Deeley, MS, RNC; Linda Rees; Fabienne Sprenger; Wolfgang Oertel, MD; Elisabeth Lang; Holly Shill, MD, Sanja Obradov, BA; Hubert Fernandez, MD, Adrienna Winters, BS; Daniela Berg, MD, Katharina Gauss; Douglas Galasko, MD, MS; Deborah Fontaine, RNCS, MS; Zoltan Mari, MD, Melissa Gerstenhaber, RNC, MSN; David Brooks, MD, Sophie Malloy, MD; Paolo Barone, MD, PhD, Katia Longo, MD, ISAB (Industry Scientific Advisory Board): Tom Comery, PhD; Bernard Ravina, MD, MSCE; Igor Grachev, MD, PhD, Kim Gallagher, PhD; Michelle Collins, PhD; Katherine L. Widnell, MD, PhD; Suzanne Ostrowizki, MD, PhD, Paulo Fontoura, MD, PhD, F. Hoffmann La-Roche; Tony Ho, MD, Johan Luthman, DDS, PhD; Marcel van der Brug, PhD; Alastair D. Reith, PhD; Peggy Taylor, ScD.
